# Interleukin-13 receptor α2 is a novel marker and potential therapeutic target for human melanoma

**DOI:** 10.1038/s41598-019-39018-3

**Published:** 2019-02-04

**Authors:** Hayato Okamoto, Yasuhiro Yoshimatsu, Taishi Tomizawa, Akiko Kunita, Rina Takayama, Teppei Morikawa, Daisuke Komura, Kazuki Takahashi, Tsukasa Oshima, Moegi Sato, Mao Komai, Katarzyna A. Podyma-Inoue, Hiroaki Uchida, Hirofumi Hamada, Katsuhito Fujiu, Shumpei Ishikawa, Masashi Fukayama, Takeshi Fukuhara, Tetsuro Watabe

**Affiliations:** 1 Laboratory of Oncology, School of Life Sciences, Tokyo University of Pharmacy and Life Sciences, Tokyo, Japan; 2 Department of Biochemistry, Graduate School of Medical and Dental Sciences, Tokyo Medical and Dental University (TMDU), Tokyo, Japan; 3 Department of Pathology, Graduate School of Medicine, The University of Tokyo, Tokyo, Japan; 4 Department of Genomic Pathology, Medical Research Institute, Tokyo Medical and Dental University (TMDU), Tokyo, Japan; 5 Department of Cardiovascular Medicine, Graduate School of Medicine, The University of Tokyo, Tokyo, Japan; 6 Project Division of Cancer Biomolecular Therapy, The Institute of Medical Science, The University of Tokyo, Tokyo, Japan; 7 Department of Advanced Cardiology, Graduate School of Medicine, The University of Tokyo, Tokyo, Japan; 8 Department of Neurology, Juntendo University School of Medicine, Tokyo, Japan

## Abstract

Malignant melanoma is one of the untreatable cancers in which conventional therapeutic strategies, including chemotherapy, are hardly effective. Therefore, identification of novel therapeutic targets involved in melanoma progression is urgently needed for developing effective therapeutic methods. Overexpression of interleukin-13 receptor α2 (IL13Rα2) is observed in several cancer types including glioma and pancreatic cancer. Although IL13Rα2 is implicated in the progression of various types of cancer, its expression and roles in the malignant melanoma have not yet been elucidated. In the present study, we showed that IL13Rα2 was expressed in approximately 7.5% melanoma patients. While IL13Rα2 expression in human melanoma cells decreased their proliferation *in vitro*, it promoted *in vivo* tumour growth and angiogenesis in melanoma xenograft mouse model. We also found that the expression of amphiregulin, a member of the epidermal growth factor (EGF) family, was correlated with IL13Rα2 expression in cultured melanoma cells, xenograft tumour tissues and melanoma clinical samples. Furthermore, expression of amphiregulin promoted tumour growth, implicating causal relationship between the expression of IL13Rα2 and amphiregulin. These results suggest that IL13Rα2 enhances tumorigenicity by inducing angiogenesis in malignant melanoma, and serves as a potential therapeutic target of malignant melanoma.

## Introduction

Malignant melanoma (melanoma) is the most aggressive type of skin cancer with high invasive and metastatic properties^1^. Much effort has been paid to develop molecular target drugs for melanoma aiming the inhibition of BRAF and MEK^2–4^, but those approaches still encounter problems of side effects^5–7^. Despite recent progress in immunotherapy^8^, there is an urgent need to develop more effective melanoma treatments being less harmful to normal cells. For this purpose, identification of new tumour markers specifically expressed in malignant melanoma will be of great importance.

We previously developed a screening method for selecting monoclonal antibodies that are recognised and internalised by target cells. Through the screening employing A375 malignant melanoma cells, we have identified antibodies that recognised interleukin-13 receptor α2 (IL13Rα2: encoded by *IL13RA2*)^9^. Several studies have demonstrated that IL13Rα2 is specifically expressed in multiple types of cancer^10^. IL-13 is one of the anti-inflammatory cytokines produced by activated CD4^+^T cells, mast cells, macrophages or dendritic cells^11^. There are two major IL-13 receptors, IL13Rα1 and IL13Rα2. IL-13 binds to its receptors in various combinations of receptor subtypes. The binding of IL-13 to its low-affinity receptor IL13Rα1 results in the activation of Janus kinase/signal transducers and activators of transcription (JAK/STAT) signalling pathway^12^. In contrast, IL13Rα2, a high-affinity receptor for IL-13, has been considered to function as a decoy receptor for IL-13-mediated signalling pathways, because it has a short intracellular domain, lacking signalling kinase part^13,14^. On the other hand, there have been also reports suggesting IL13Rα2 to play a role in IL-13 signalling^15,16^. Recently, Newman and colleagues reported that IL13Rα2 cooperated with epidermal growth factor receptor mutant (EGFRvIII) to activate the ERK and STAT3 pathways to promote the progression of glioblastoma multiforme (GBM)^17^, suggesting that IL13Rα2 actively participates in multiple signal transduction pathways.

A correlation between the expression level of IL13Rα2 and increased invasiveness as well as metastatic potential of human glioma^18,19^, breast cancer^20^ and pancreatic cancer^21^ have been reported. Among normal tissues, IL13Rα2 can be found only in spermatocytes^22^, suggesting that IL13Rα2 can be considered as a good candidate for developing new targeted therapeutics that potentially would exhibit fewer side effects. In fact, by taking advantage of the high affinity of IL13Rα2 for IL-13 as well as its cancer cell-specific expression, several therapeutic agents targeting IL13Rα2 have been designed. For example, glioma-targeted therapy based on the delivery of chimeric protein comprising IL-13 and *Pseudomonas* exotoxin A (PE), has been already under development^19^. While the expression of IL13Rα2 in melanoma has been also reported^23^, its expression profile and roles in melanoma progression remain to be elucidated. Thus in the present study, we studied the expression pattern of IL13Rα2 in malignant melanoma and elucidated the relationship between the expression of IL13Rα2 and tumour progression in melanoma.

## Results

### IL13Rα2 is highly expressed in a subgroup of patients with melanoma

We previously reported that A375 melanoma cells were recognised by anti-IL13Rα2 antibodies^9^. To examine the relative level of IL13Rα2 expression in melanoma cells, Cancer Cell Line Encyclopedia (CCLE) was used to analyse the frequency of *IL13RA2* expression in various carcinoma cell lines. As shown in Fig. S1, *IL13RA2* was highly expressed in some melanoma cell lines, suggesting that IL13Rα2 is highly expressed in certain regions of melanoma.

**Figure 1:**
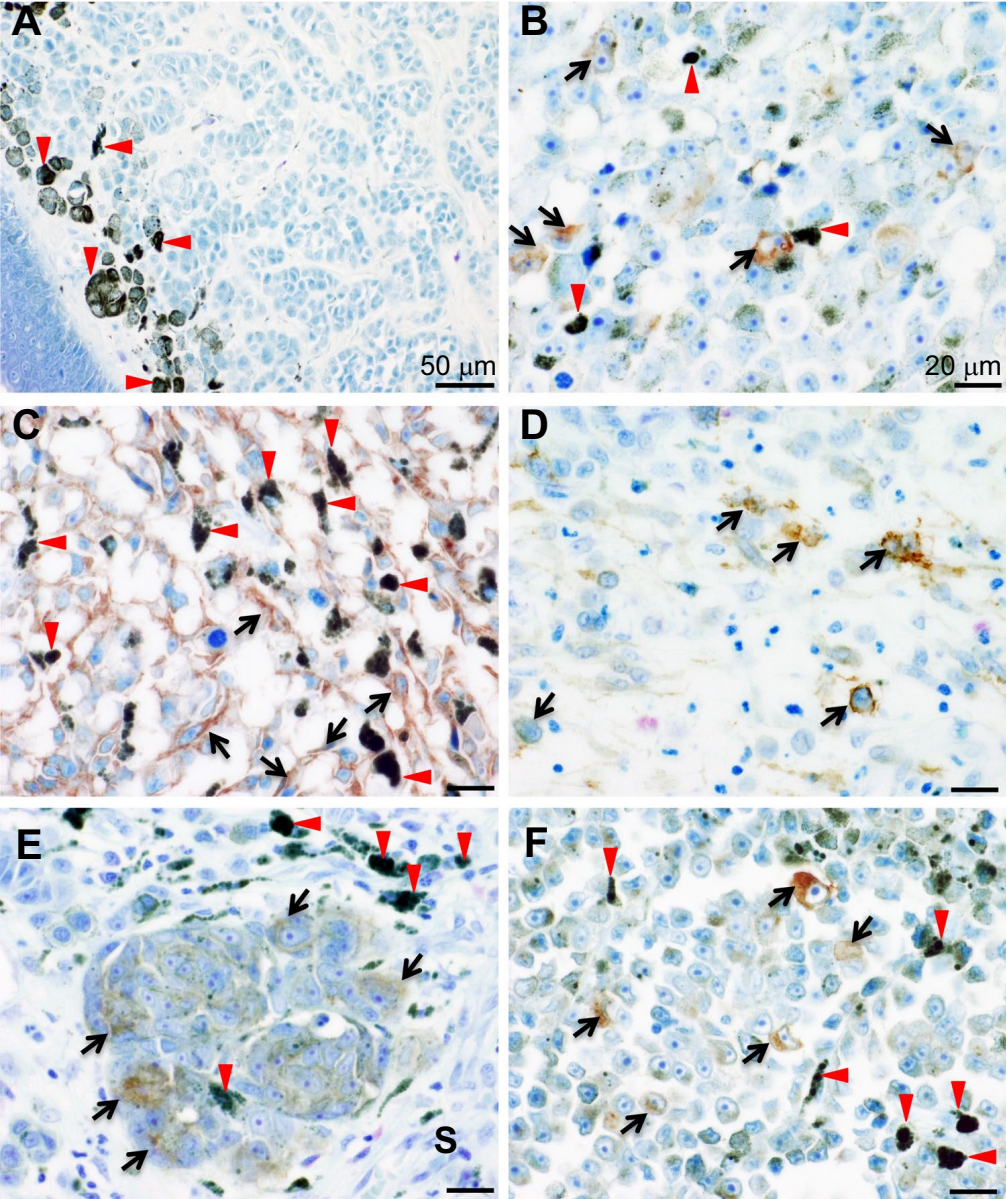
Tissue microarray analyses for IL13Rα2 expression. Multiple series of tissue microarrays were subjected to immunohistochemical analysis by using anti-IL13Rα2 antibody (KH7B9). Expression of IL13Rα2 was detected in the cytoplasm or membrane of melanoma cells (arrows). Red arrowheads indicate melanin pigment. (**A**) Benign naevus of the right face. (**B**) Metastatic malignant melanoma from the armpit (lymph node). (**C**) Malignant melanoma of the thigh. (**D**) Malignant melanoma of the cunnus. (**E**) Malignant melanoma of the skin. IL13Rα2 was expressed by melanoma cells (arrows) but not by stromal cells (S). (**F**) Malignant melanoma of the right sole. Scale bar: 50 μm (**A**), 20 μm (**B**–**F**).

Next we examined the frequency of IL13Rα2 expression in human melanoma samples by using tissue microarrays. Our immunohistochemical analysis by using anti-IL13Rα2 antibody (KH7B9), detected IL13Rα2 in the xenograft tumour cells derived from A375, but not in IL13Rα2-negative cells (A375-IL13RA2 KO and A2058 cells) (Fig. S2A–C), thus confirming the specificity of the KH7B9. In addition, in agreement to the previous report, among normal human tissues, the signal corresponding to IL13Rα2 was only detected in spermatocytes^22^ (Fig. S2D–H). Moreover, IL13Rα2 expression was not detected in normal skin or benign naevus specimens (Fig. 1A). On the other hand, our data showed that substantial expression of IL13Rα2 was observed in various human melanoma tissues including metastatic malignant melanoma from the armpit (lymph node) (Fig. 1B), malignant melanoma from the thigh (Fig. 1C), cunnus (Fig. 1D), skin (Fig. 1E) and right sole (Fig. 1F). Positive staining for IL13Rα2 expression was detected in 14 samples (12 primary tumours; 2 metastatic tumours) out of 187 independent human melanoma samples (137 primary tumours; 50 metastatic tumours), which corresponded to 7.5% (14/187) of total cases examined, suggesting that IL13Rα2 was expressed in a group of human melanoma. IL13Rα2 staining pattern varied among tumour tissue samples examined (Supplementary Table 1) with IL13Rα2 staining observed in >90% tumour cells in a tumour tissue sample obtained from one patient (Fig. 1C). However, IL13Rα2 expression was observed only in a subset of tumour cells (≤10% tumour cells) in >50% tissue samples showing positive IL13Rα2 staining (Fig. 1B,D–F and Supplementary Table 1). No significant difference was observed in the rate of positive IL13Rα2 staining between the primary and metastatic tumour tissue samples examined (Supplementary Table 1). These expression profiles suggested that IL13Rα2 is a novel cancer-testis antigen.

### IL13Rα2 suppresses *in vitro* proliferation of SK-MEL-28 melanoma cells

**Figure 2:**
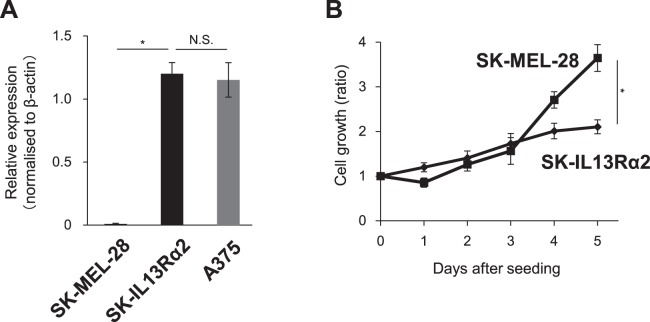
Effects of IL13Rα2 expression on the *in vitro* proliferation of SK-MEL-28 melanoma cells. The SK-MEL-28 cells were transfected with the expression plasmid encoding IL13Rα2 to establish SK-IL13Rα2 to study the effect of IL13Rα2 on cell proliferation. (**A**) The expression of IL13Rα2 in the SK-MEL-28, SK-IL13Rα2 and A375 (IL13Rα2-positive) cells was determined by qRT-PCR. (**B**) 2 × 10^4^ of SK-MEL-28 and SK-IL13Rα2 cells were seeded into 6-well plates and were allowed to grow for 5 days. The cells were harvested and were counted on the indicated day. All values are shown as the ratios to the number of the seeded cells on day 0 and are mean ± SD. *p < 0.05; Student’s t-test.

It is well known that different tumour cells show distinct morphological and phenotypic profiles, including cell morphology, gene expression and cell proliferation rate, which is termed tumour heterogeneity. Our finding revealing that IL13Rα2 expression was restricted to only a subset of melanoma cells prompted us to examine whether IL13Rα2 could regulate the oncogenic capacity of malignant melanoma. In order to address this issue, we used malignant melanoma SK-MEL-28 cells which show no detectable expression of IL13Rα2 (Fig. 2A). The SK-MEL-28 cells were transfected with an IL13Rα2 expression vector followed by quantitative RT-PCR (qRT-PCR). IL13Rα2 stable transfectants, SK-IL13Rα2 cells, showed similar expression level of IL13Rα2 compared to that of A375 melanoma cells. In order to study the effect of IL13Rα2 expression on the proliferation of malignant melanoma cells, the SK-MEL-28 cells and SK-IL13Rα2 cells were allowed to grow for 5 days and the number of cells was determined *in vitro*. We found that the expression of IL13Rα2 was inversely correlated with the proliferative activity of melanoma cells, because the SK-IL13Rα2 cells, expressing IL13Rα2 exhibited decreased cell growth rate compared with the parental SK-MEL-28 cells (Fig. 2B), suggesting that IL13Rα2 may play an inhibitory role in *in vitro* proliferation of melanoma cells. This inhibitory effect of IL13Rα2 on growth of SK-MEL-28 cells seem not to be mediated by the IL-13-related signals because the addition of IL-13 did not affect the proliferation of the SK-MEL-28, SK-IL13Rα2 or A375 cells (Fig. S3).

### Size of tumours formed by SK-MEL-28 melanoma cells increased upon IL13Rα2 expression

**Figure 3:**
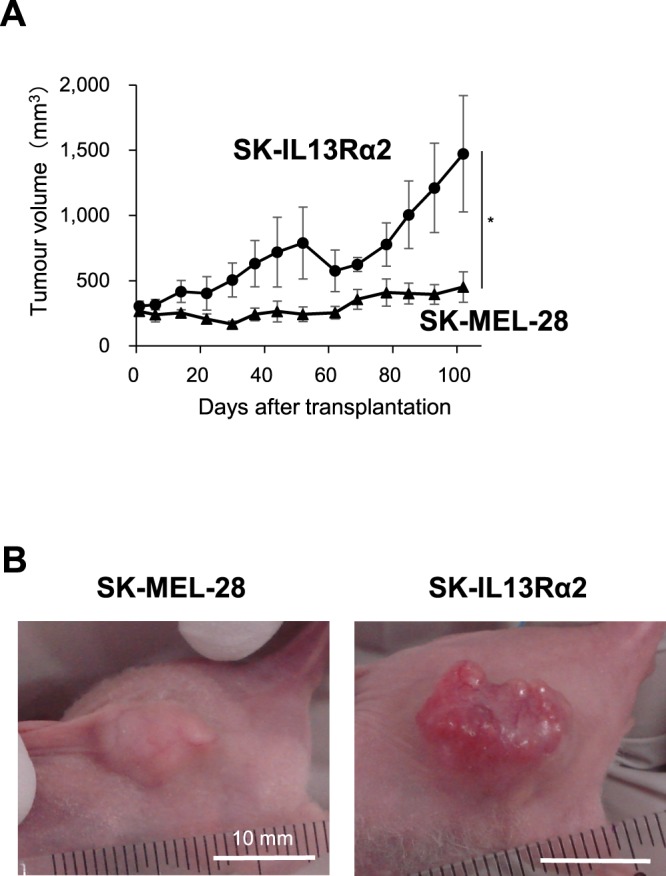
Effects of IL13Rα2 expression on the *in vivo* tumour formation of SK-MEL-28 melanoma cells. The SK-MEL-28 (n = 6) and SK-IL13Rα2 (n = 6) cells were subcutaneously transplanted into the immunodeficient mice. (**A**) Tumour growth was assessed for 102 days after transplantation by callipers and was calculated from minor axis and major radius. All values are mean ± SE. *p < 0.05; Student’s t-test. (**B**) Images of representative tumours at 102 days post-transplantation. Scale bar: 10 mm.

Because IL13Rα2 decreased the *in vitro* proliferation of melanoma cells, we next attempted to study the effect of IL13Rα2 expression on *in vivo* tumour growth of melanoma cells. The SK-IL13Rα2 or SK-MEL-28 cells were subcutaneously xenografted into immunodeficient mice, followed by a measurement of formed tumours. In contrast to the *in vitro* data, tumours derived from the SK-IL13Rα2 cells were larger than those formed by parental SK-MEL-28 cells (Fig. 3A), suggesting that high level of IL13Rα2 expression promoted tumorigenicity *in vivo*.

### Expression of IL13Rα2 stimulated vessel formation in SK-MEL-28 cell-derived tumours

**Figure 4:**
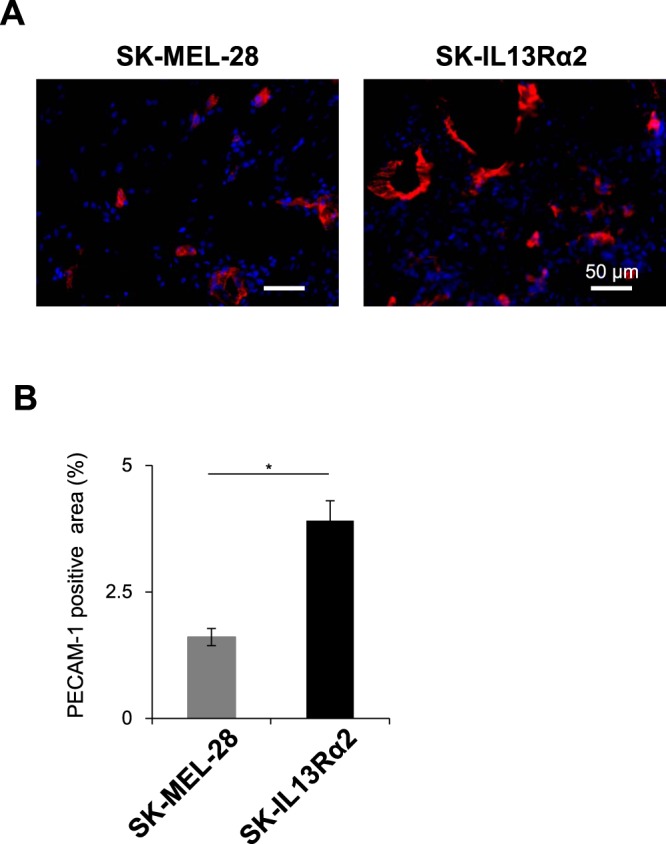
Effects of IL13Rα2 expression on tumour angiogenesis. (**A**) Sections of the tumours derived from the SK-MEL-28 (n = 5) and SK-IL13Rα2 (n = 6) cells were subjected to immunofluorescence staining with the anti-PECAM-1 antibodies. Scale bar: 50 μm. (**B**) PECAM-1 positive area represented as a fraction of the total image area. All values are mean ± SE. *p < 0.05; Student’s t-test.

Although IL13Rα2 suppressed *in vitro* proliferation of the SK-MEL-28 cells (Fig. 2B), the tumours formed by the SK-IL13Rα2 cells grew faster than those derived from the SK-MEL-28 cells (Fig. 3A). This result suggested that IL13Rα2 may specifically promote tumour growth only while being expressed by the cells residing within tumour microenvironment (TME). The TME is composed of not only cancer cells, but also other components such as blood vessels and fibroblasts, which also actively participate in tumour formation. Especially blood vessels play an essential role in promoting growth of tumour tissue by supplying oxygen and nutrients necessary for the growth of tumour. Interestingly, the tumour mass formed by the SK-IL13Rα2 cells had more reddish appearance than the tumour mass derived from the control SK-MEL-28 cells (Fig. 3B), implying that the formation of large tumour mass by SK-IL13Rα2 may depend on enhanced angiogenesis. In order to study whether IL13Rα2-dependent tumour growth was mediated by enhanced angiogenesis, we investigated whether altered expression of IL13Rα2 would affect new vessel formation in melanoma tumour tissues. As shown in Figs 4 and S4, the PECAM-1-positive vascular area in the tumour tissue derived from the SK-IL13Rα2 cells was significantly larger when compared with the tumour derived from the SK-MEL-28 cells, implying that the expression of IL13Rα2 in malignant melanoma enhanced angiogenesis.

### Endogenous IL13Rα2 expression in A375 melanoma cells is indispensable for *in vivo* tumour formation and angiogenesis

**Figure 5:**
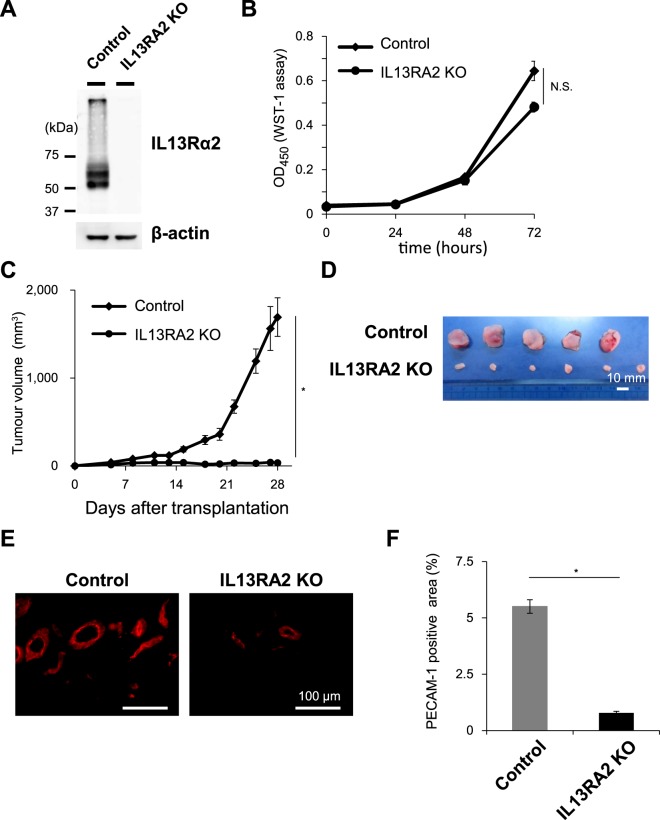
Roles of IL13Rα2 in the *in vivo* tumour formation and angiogenesis in A375 xenograft model. *IL13RA2* gene was knocked out in A375 melanoma cells. (**A**) The expression of IL13Rα2 in A375-Control and A375-IL13RA2 KO cells was determined by immunoblotting analysis with the anti-IL13Rα2 (KH7B9) and anti-β-actin antibodies. Cropped images from the same blots are shown. Full-length blots are presented in Supplementary Fig. S7. (**B**) In all, 3 × 10^3^ of A375-Control (n = 6) and A375-IL13RA2 KO (n = 6) cells were seeded into 12-well plates and allowed to grow for 3 days. Cells were harvested and were counted at the indicated period. (**C**) The A375-Control and A375-IL13RA2 KO cells were subcutaneously transplanted into immunodeficient mice. Tumour growth was measured using callipers and was calculated from minor axis and major radius. (**D**) Images of representative tumours. Scale bar: 10 mm. (**E**) Sections of tumours derived from A375 Control (n = 5) and A375-IL13RA2 KO (n = 6) cells were subjected to immunofluorescence staining with the anti-PECAM-1 antibodies. Scale bar: 100 μm. (**F**) PECAM-1 positive area represented as the fraction of total image area. All values are mean ± SE. *p < 0.05; Student’s t-test.

Since we found that increased level of IL13Rα2 expression in the SK-MEL-28 cells promoted *in vivo* tumorigenicity and angiogenesis (Fig. 3), we next questioned whether loss of endogenous IL13Rα2 expression in A375 cells would affect *in vivo* tumour growth. For this purpose we generated A375-IL13RA2 knockout (KO) cells using CRISPR-Cas9 regulated by transcription and nuclear-shuttling (CRONUS) system, a recently developed, inducible CRISPR-Cas9 system^24^ (Figs 5A and S7). Although the loss of IL13Rα2 expression in A375 cells did not affect *in vitro* proliferation (Fig. 5B), tumours formed after the subcutaneous inoculation of the A375-IL13RA2 KO cells were significantly smaller than those derived from the control cells (Fig. 5C,D), suggesting that endogenous IL13Rα2 expression in A375 melanoma cells is necessary for *in vivo* tumour formation. In addition, a detailed analysis of the tumour tissues derived from the A375-IL13RA2 KO cells showed a dramatic decrease in the number of PECAM-1-positive vessels (Fig. 5E,F) indicating that new vessel formation was strongly affected by the absence of IL13Rα2 expression and revealing it to be indispensable for A375 tumour formation and angiogenic events.

### IL13Rα2 increases amphiregulin expression in various types of melanoma cells

Our findings revealed that IL13Rα2 accelerated tumour growth *in vivo* not by promoting cell proliferation but rather by affecting *in vivo* tumour angiogenesis. Therefore, we hypothesised that IL13Rα2 in malignant melanoma would stimulate the secretion of angiogenesis-inducing factors. In order to determine the molecules involved in IL13Rα2-mediated angiogenesis in melanoma, we performed Angiogenesis Antibody Array using the lysates of tumour tissues derived from xenografted SK-MEL-28 and SK-IL13Rα2 cells (unpublished data) and identified multiple angiogenesis-related molecules including amphiregulin.

**Figure 6:**
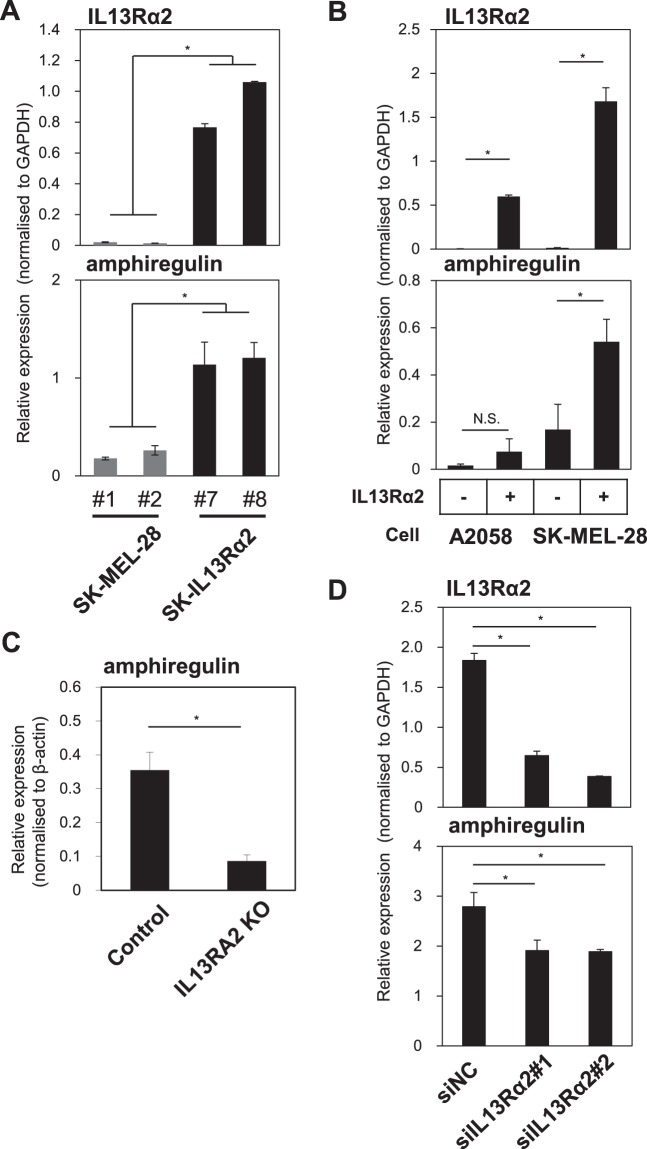
Effects of IL13Rα2 on amphiregulin expression in various types of melanoma cells. (**A**) Total RNAs were prepared from tumour tissues derived from the SK-MEL-28 (#1, #2) and SK-IL13Rα2 (#7, #8) cells and were subjected to quantitative RT-PCR analyses for the expression of IL13Rα2 (top) and amphiregulin (bottom). (**B**) The A2058 and SK-MEL-28 melanoma cells were transfected with IL13Rα2 expression vector, followed by qRT-PCR analysis for the expression of IL13Rα2 (top) and amphiregulin (bottom). (**C**) *IL13RA2* was knocked out in the A375 melanoma cells, followed by qRT-PCR analysis for the expression of IL13Rα2. (**D**) The A375 melanoma cells were transfected with negative control (NC) siRNAs or siRNAs for IL13Rα2 (#1 and 2), followed by qRT-PCR analysis for the expression of IL13Rα2 (top) and amphiregulin (bottom). All values are mean ± SD. *p < 0.05; Student’s t-test.

Amphiregulin (encoded by *AREG*) is a member of the epidermal growth factor (EGF) family, and is known to promote the growth of multiple types of cells^25^. In order to determine whether expression of amphiregulin transcript is also upregulated by IL13Rα2, we performed qRT-PCR analyses for the expression of IL13Rα2 and amphiregulin using the cDNAs prepared from tumours originated from SK-MEL-28 and SK-IL13Rα2 cells. As shown in Fig. 6A, the level of amphiregulin expression was increased in the tumours originated from the SK-IL13Rα2 cells as compared with those from the SK-MEL-28 cells, suggesting a positive correlation between IL13Rα2 and amphiregulin expressions.

In addition, in order to investigate whether the increased expression of amphiregulin in melanoma cells is cell-autonomously regulated by IL13Rα2, we studied the amphiregulin expression in various melanoma cells in which IL13Rα2 expression was altered. Increased expression of IL13Rα2 in A2058 and SK-MEL-28 melanoma cells, which do not express endogenous IL13Rα2, the expression of amphiregulin was upregulated (Fig. 6B). In contrast, when *IL13RA2* was deleted in A375 cells, amphiregulin expression was significantly decreased (Fig. 6C). To confirm the effects of loss-of-function of IL13Rα2 on amphiregulin expression, the A375 melanoma cells were subjected to RNA silencing using siRNA specific for IL13Rα2. Downregulation of IL13Rα2 expression in the A375 cells resulted in decreased level of amphiregulin expression (Fig. 6D) thus confirming a positive correlation between IL13Rα2 and amphiregulin expressions. These results indicate that the level of IL13Rα2 expression regulates mRNA transcription of amphiregulin.

Furthermore, in order to determine whether *IL13RA2* expression level in melanoma clinical samples was correlated with *AREG* expression, we analysed *IL13RA2* and *AREG* expression by using the TCGA (The Cancer Genome Atlas) database. In accordance with the present *in vitro* and *in vivo* analyses in various melanoma cells, TCGA analysis showed that *IL13RA2* expression was correlated with *AREG* expression in malignant melanoma patients (Fig. S5), suggesting that IL13Rα2 regulated amphiregulin expression in human melanoma patients.

### Size of tumours derived from SK-MEL-28 cells is positively regulated by amphiregulin expression

**Figure 7:**
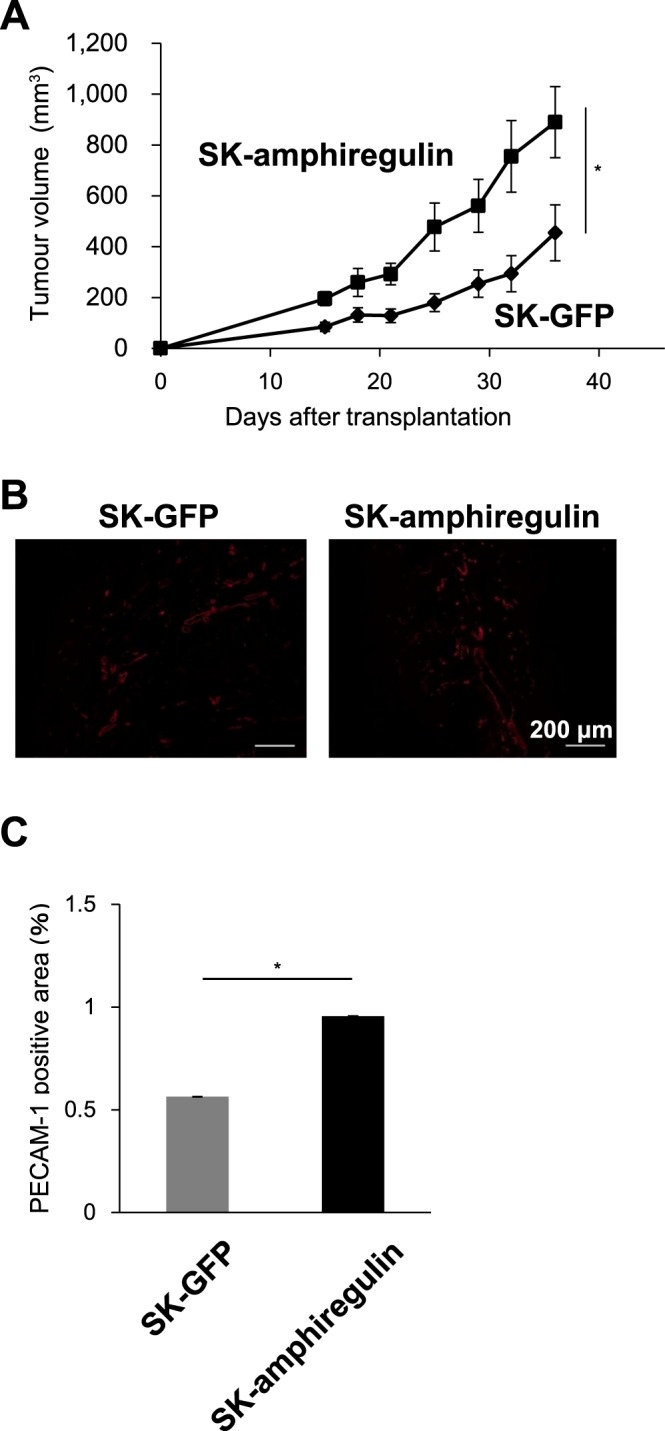
Effects of amphiregulin expression on the *in vivo* tumour formation and angiogenesis in the SK-MEL-28 xenograft model. The SK-MEL-28 cells were transfected with the expression plasmid encoding amphiregulin to establish SK-amphiregulin cells. The SK-amphiregulin and SK-GFP cells, were then subcutaneously transplanted into the immunodeficient mice, followed by the measurement of tumour size (**A**). (**B**) Sections of the tumours derived from the SK-GFP (n = 6) and SK-amphiregulin (n = 6) cells were examined by performing immunofluorescence staining with the anti-PECAM-1 antibodies. Scale bars, 200 μm. (**C**) PECAM-1 positive area represented as the fraction of the total image area. All values are mean ± SE. *p < 0.05; Student’s t-test.

We showed that expression of IL13Rα2 in the malignant melanoma SK-MEL-28 cells increased expression of angiogenic factor, amphiregulin as well as enhanced tumour angiogenesis and tumorigenicity *in vivo*. However, it remained to be clarified whether expression of amphiregulin enhanced the tumorigenicity of malignant melanoma. Therefore, we investigated whether amphiregulin augmented the progression of malignant melanoma *in vivo* by using SK-MEL-28 cells overexpressing amphiregulin, SK-amphiregulin cells. The tumours originated from the SK-amphiregulin cells were significantly larger than those formed by control cells, SK-GFP (Fig. 7A). To study whether amphiregulin-dependent tumour growth was mediated by enhanced angiogenesis, we investigated whether altered amphiregulin expression would affect new vessel formation in melanoma tumour tissues. We observed that PECAM-1-positive vascular area in the tumours derived from the SK-amphiregulin cells was significantly larger than that in the tumours derived from SK-GFP cells, implying that amphiregulin expression in malignant melanoma enhanced angiogenesis. These results suggest that expression of amphiregulin in malignant melanoma promotes tumour growth by inducing tumour angiogenesis.

## Discussion

In this study we investigated the expression of IL13Rα2 in malignant melanoma and its role in the progression of melanoma. Our data revealed that the expression of IL13Rα2, which was detected in a subgroup of human melanoma specimens, was correlated with the tumorigenicity of multiple types of melanoma cells. While high level of IL13Rα2 expression inhibited cell proliferation *in vitro*, it also promoted the expression of amphiregulin, a proangiogenic factor, which in turn resulted in enhanced angiogenesis and increased tumour formation *in vivo*.

We showed that IL13Rα2 in malignant melanoma promoted tumorigenicity by stimulating angiogenesis using gain-of-function (Figs 3 and 4) and loss-of-function (Fig. 5) studies. Previous reports showed that the expression of IL13Rα2 in other types of cancers had been associated with the progression of cancer by promoting metastatic and invasive abilities of cancer cells^17,18,20^. Although the role of IL13Rα2 in melanoma progression is likely attributed to its proangiogenic function, its roles in other aspects of melanoma progression such as metastasis need to be further characterised.

The present study indicated that IL13Rα2 expression in melanoma cells enhanced tumour angiogenesis (Figs 4 and 5). Although IL13Rα2 expression in the SK-MEL-28 cells slightly upregulated the expression of vascular endothelial growth factor (VEGF), a well-known tumour angiogenic factor, the upregulation can be considered minimal (1.5-fold), compared to that of amphiregulin (3.2-fold) (Figs 6 and S6). The loss of IL13Rα2 expression in the A375 melanoma cells did not alter the VEGF expression (Fig. S6). Instead, our comprehensive analysis using Angiogenesis Antibody Array revealed that the expression of IL13Rα2 was positively correlated with the enhanced expression of amphiregulin (Fig. 6), but not that of VEGF (unpublished data). Amphiregulin is a member of the EGF family. Its binding to the EGFR activates intracellular signals leading to various physiological responses including enhanced cell proliferation and cardiac hypertrophy^25,26^. Elevated expression of amphiregulin has been detected in various types of cancers^25^ where in addition to promoting the proliferation of tumour cells, it also induces tumour vascularisation^27^. Consistent with these reports, we showed that elevated expression of amphiregulin promoted tumorigenesis of melanoma (Fig. 7). Our results, taken together with the previous reports, suggest that the expression of IL13Rα2 enhances melanoma tumorigenesis *in vivo* via promoting angiogenesis through the expression of angiogenesis-triggering factors such as amphiregulin.

We showed that IL13Rα2 expression not only enhanced the expression of amphiregulin (Fig. 6) but also suppressed *in vitro* proliferation of SK-MEL-28 cells (Fig. 2), suggesting that IL13Rα2 transduces intracellular signals that regulated these events in melanoma cells. IL-13 did not affect the proliferation of the SK-MEL-28 or A375 melanoma cells in the present study (Fig. S3); however, previous study in a colorectal cancer epithelial cell line HT-29 revealed that IL-13 signal interfered with cell proliferation by inducing cell death^28^. While binding of IL-13 to IL13Rα1 results in recruitment of interleukin 4 receptor (IL4R), followed by formation of IL13Rα1-IL4R complex and activation of intracellular signals^12^, these effects of IL-13 can be mediated independently of IL13Rα1-IL4R signalling axis because the IL-13-induced injury-related responses in the mouse intestinal mucosa take place even under IL4R deficiency^29^. The interaction of IL13Rα2 with EGFRvIII induces progression of GBM by activating intracellular signalling^17^. However, detailed mechanisms how IL13Rα2 regulates the expression of amphiregulin and proliferation of melanoma cells need to be elucidated in the future.

Melanin affects various cellular processes both in normal melanocytes and melanoma cells as well as in their surrounding microenvironment^30^. Moreover, melanin level is positively correlated with melanoma aggressiveness and radiotherapy resistance^31,32^. Melanogenesis induction in melanoma cells upregulates HIF-1α-related pathways, including stress response-related pathways, glucose metabolism and angiogenesis^33,34^. In the present study we used multiple melanoma cell lines, which are characterised by different level of melanin content. A375 cells are amelanotic melanoma cells, SK-MEL-28 cells are lightly pigmented, while A2058 cells represents pigmented melanoma cell line. No obvious correlation was observed between expression of IL13Rα2 and pigmentation in the melanoma cells used in the present study. However, the role of IL13Rα2 with respect to the pigmentation in the pathogenesis and progression of melanoma should be elucidated in the future.

Our previous study suggested that IL13Rα2 could be a novel biomarker for malignant melanoma^9^. IL-13-related pathway has been already targeted while developing drug for gliomas. The approach employs a chimeric protein composed of IL-13 and PE that exploits the high affinity to IL13Rα2^21^. We previously reported functional antibodies that targeted IL13Rα2 and became internalised by targeted cells^9^. These anti-IL13Rα2 antibodies would facilitate development of antibody-drug conjugates, drugs developed by conjugation of an anticancer agent to a monoclonal antibody. The present finding that IL13Rα2 is expressed in a subgroup of melanoma cells and promotes tumorigenesis suggest IL13Rα2 to be a promising target for the development of novel therapeutic strategy against melanoma.

## Materials and Methods

### Cells and cell culture

Human melanoma A375, SK-MEL-28 and A2058 cells were obtained from American Type Culture Collection (ATCC), RIKEN Cell Bank and the Japanese Collection of Research Bioresource, respectively. The A375 and A2058 cells were maintained in DMEM; high glucose (4.5 g/L, Nacalai Tesque, Kyoto, JAPAN) supplemented with 10% fetal calf serum (FCS, Sigma-Aldrich, St. Louis, MO, USA), 100 units/mL penicillin and 100 μg/mL streptomycin. The SK-MEL-28 cells were maintained in Eagle-modified MEM (E-MEM, Wako, Saitama, JAPAN) supplemented with 10% FCS (Sigma-Aldrich), 1 mM sodium pyruvate (Nacalai Tesque), 100 units/mL penicillin and 100 μg/mL streptomycin. All the cells were grown in humidified incubator at 37 °C and 5% CO_2_.

### Generation of anti-IL13Rα2 monoclonal antibody (KH7B9)

The anti-IL13Rα2 antibodies were generated by immunising mice with denatured recombinant human IL13Rα2, which yielded monoclonal antibody KH7B9. The antibody was purified from hybridoma culture supernatant using Prosep-A (Merck Millipore, Burlington, MA, USA) according to the instructions of the manufacturer.

### Immunohistochemistry

Human melanoma tissue microarray slides, ME208, ME1002a, and ME1004e, were purchased from US Biomax (Rockville, MD, USA). ME208 consisted of 208 cores representing 69 cases in triplicate: 30 were primary melanoma tissues, 30 were metastatic melanoma tissues and 9 were normal skin tissues. ME1002a consisted of 100 cores representing 50 cases in duplicate: 45 were malignant melanoma, 1 was cancer adjacent a normal skin tissue, 4 were normal skin tissues. ME1004e consisted of 100 cores representing 100 cases (single core per case): 62 were malignant melanoma, 20 were metastatic malignant melanoma and 18 were naevus tissues. All core diameters were 1 mm. Immunostaining for IL13Rα2 (by using KH7B9 antibody, 1:200) was performed according to standard protocols using an autostainer (BenchMark XT; Ventana Medical Systems, Inc., Tucson, AZ, USA). Sections were counterstained with 2% Giemsa solution (Sigma-Aldrich). The Giemsa solution changes the melanin pigment to green, which facilitates discrimination from brown positive signals^35^. IL13Rα2 immunostaining was considered positive when at least 1% of tumour cells showed cytoplasmic or membranous staining. Immunofluorescent staining of human melanoma xenografts was done as described previously^36^. Briefly, the formation of blood vessels in xenografted tumours were evaluated after the tumours were harvested on day 102 (Fig. 4), day 28 (Fig. 5) and day 53 (Fig. 7) by immunostaining using anti-platelet and endothelial cell adhesion molecule (PECAM-1) antibodies (BD Biosciences, San Jose, CA, USA, cat. no. 55370, 1:500).

### RNA isolation and quantitative RT-PCR

Total RNA was prepared with RNeasy reagent (QIAGEN, Hilden, Germany) and was reverse transcribed by random priming and PrimeScript II 1st strand cDNA Synthesis Kit (Takara Bio, Otsu, JAPAN). Quantitative RT-PCR analysis was performed using the GeneAmp 5700 Sequence Detection System and StepOne Plus Real-Time PCR System (Applied Biosystems/Thermo Fisher Scientific, Waltham, MA, USA). All expression data were normalised using the expression of GAPDH or β-actin gene. The primer sequences used for analysis are listed in Supplementary Table 2.

### Cell proliferation assay

The cells were seed into 6-well plate and allowed to grow for an indicated period of time, followed by direct cell counting with hemocytometer or WST-1 assay by measuring absorbance of reduced formazan at OD_450_. The experiments were performed in quintuplicate (direct cell counting) or duplicate (WST-1 assay).

### Construction of lentiviral vectors and infection

The cDNAs encoding *IL13RA2* and *AREG* were cloned from A375 cells and were subcloned into pCSII-EF-RfA lentivirus vectors as described previously^36^. 293FT cells were co-transfected with the expression plasmids and packaging plasmids (pCMV-VSV-G-RSV-Rev and pCAG-HIVgp). The viral supernatants were collected 72 hours after transfection. SK-MEL-28 cells (5.0 × 10^4^ cells/well in 6-well tissue culture plates) were infected with lentiviral particles resulting in the SK-IL13Rα2, SK-amphiregulin or SK-GFP cells (negative control). All the experiments were approved by “the Safety Control Committee for Experiments Using Genetically Modified Organisms, Etc.” and done according to the guideline of “Safety Control Regulations for Experiments Using Genetically Modified Organisms, Etc., Tokyo Medical and Dental University” (registration number: 2016-032C8).

### Establishment of A375 cells deficient for *IL13RA2* gene using CRONUS system

*IL13RA2* knockout cells were established using CRONUS (CRISPR-Cas9 regulated by transcription and nuclear-shuttling) system as described previously^24^. A gRNA target sequence for IL13RA2; 5′-GAGAGATAACCTAAGTATCCTGG-3′ (PAM sequence underlined) was designed with CRISPRdirect (https://crispr.dbcls.jp/). The primer sequences used for cloning are as follows: sgRNA-IL13RA2-127/149-fwd primer; 5′- GAGACCACTTGGATCCGAGAGATAACCTAAGTATCCGTTTTAGAGCTAGAAATAGCA (underlined sequence corresponds to gRNA spacers), sgRNA-Universal-rev primer; 5′-GCCCGGGTTTGAATTCAAAAAAAGCACCGACTCGGTGCCACTTTTTCAAGTTGATAACGGACTAGCCTTATTTTAACTTGCTATTTCTAGCTCTAA-3′. Stable transfectants of A375 melanoma cells were established by transfection with the plasmids described previously^24^, and selection by combined puromycin and hygromycin treatment. The induction of Cas9 expression and its nuclear translocation was achieved by incubating resistant cells with a mixture of 5 μM doxycycline (LKT Laboratories, St. Paul, MN, USA) and 10 μM dexamethasone (Wako) for 5 days. A375-IL13RA2 KO clones were then isolated by performing limiting dilution. Effective knocking-out of *IL13RA2* was confirmed at the protein level by immunoblotting with anti-human IL13Rα2 antibody KH7B9.

### Subcutaneous xenograft melanoma model

Animal experiments were approved by the Institutional Animal Care and Use Committee at Tokyo University of Pharmacy and Life Sciences (approval number: L16-13, LS27-007) and Tokyo Medical and Dental University (registration number: A2018-210C) and were done according to the guidelines of the Animal Care Standards of both the institutes. The SK-IL13Rα2, SK-MEL-28, A375-IL13RA2 KO, A375-Control, SK-amphiregulin or SK-GFP cells were inoculated subcutaneously (s.c.) into the 6-week-old female immunodeficient nude mice by injection of 1 × 10^7^ cells of SK-MEL-28 and its derivatives, and 1 × 10^5^ cells of A375 and its derivatives in 100 μL of Matrigel (Corning, New York, NY, USA), respectively. Tumour formation was examined at the indicated time points. Tumour volume (V) was calculated using the formula: V (mm^3^) = L × W2 × 0.5, in which L corresponds to the length of the tumour in mm and W to the width of the tumour in mm, respectively.

### Immunoblotting analysis

Immunoblotting analysis was performed as described previously^37^ using antibodies to IL13Rα2 (KH7B9) and β-actin (A1978, SIGMA, St. Louis, MO, USA).

### Angiogenesis array

Upregulation of angiogenesis-related factors was determined using a Proteome Profiler™ Human Angiogenesis Antibody Array (R&D Systems, Minneapolis, MN, USA, cat. no. ARY007), which allows the identification of angiogenic factors present in tumour lysates. The array consists 59 types of nitrocellulose membrane-bound antibodies specific for various angiogenic factors. The array was conducted according to the manufacturer’s protocol. Briefly, tumour tissue extracts were prepared from xenografted tumours derived from the SK-MEL-28 and SK-IL13Rα2 cells and incubated with a cocktail of biotinylated antibodies against various angiogenic factors. The mixture was then incubated with membrane-bound anti-angiogenic factors antibodies, followed by incubation of trapped complexes with HRP-conjugated streptavidin, and detection with chemiluminescent detection reagent.

### RNA interference

Predesigned small interfering RNAs for IL13Rα2 and negative control were purchased from Invitrogen/Thermo Fisher Scientific. Obtained siRNAs (Stealth RNAi, Cat. no. 1299001 and 1330001) were introduced into cells by using RNA iMAX (Invitrogen) according to the protocol suggested by the manufacturer.

### Statistical analyses

Results were compared by Student’s t-test. Differences were considered significant when P < 0.05. All statistical tests were two sided except for animal experiments (one sided).

## Supplementary information

Supplementary Information
