# Supplementary material for: Interleukin-13 receptor α2 is a novel marker and potential therapeutic target for human melanoma
Source: Sci Rep. 2019 Feb 4;9:1281. doi: 10.1038/s41598-019-39018-3 (PMC6362032; doi:10.1038/s41598-019-39018-3)

Supplementary Information

Interleukin-13 receptor α2 is a novel marker and potential therapeutic target for human melanoma

Hayato Okamoto[1], Yasuhiro Yoshimatsu[1,2], Taishi Tomizawa[1], Akiko Kunita[3], Rina Takayama[2], Teppei Morikawa[3], Daisuke Komura[4], Kazuki Takahashi[2], Tsukasa Oshima[5], Moegi Sato[1], Mao Komai[1], Katarzyna A. Podyma-Inoue[2], Hiroaki Uchida[1,6], Hirofumi Hamada[1], Katsuhito Fujiu[5,7], Shumpei Ishikawa[4], Masashi Fukayama[3], Takeshi Fukuhara[1,8] and Tetsuro Watabe[1,2]

[1]Laboratory of Oncology, School of Life Sciences, Tokyo University of Pharmacy and Life Sciences, Tokyo, Japan,
[2]Department of Biochemistry, Graduate School of Medical and Dental Sciences, Tokyo Medical and Dental University (TMDU), Tokyo, Japan,
[3]Department of Pathology, Graduate School of Medicine, The University of Tokyo, Tokyo, Japan,
[4]Department of Genomic Pathology, Medical Research Institute, Tokyo Medical and Dental University (TMDU), Tokyo, Japan,
[5]Department of Cardiovascular Medicine, Graduate School of Medicine, The University of Tokyo, Tokyo, Japan,
[6]Project Division of Cancer Biomolecular Therapy, The Institute of Medical Science, The University of Tokyo, Tokyo, Japan,
[7]Department of Advanced Cardiology, Graduate School of Medicine, The University of Tokyo, Tokyo, Japan,
[8]Department of Neurology, Juntendo University School of Medicine, Tokyo, Japan

Materials and methods

Gene expression analysis of publicly available data

RNA-sequencing data of *IL13RA2* from the Cancer Cell Line Encyclopedia (CCLE) project were downloaded from the Broad CCLE portal and 1444 cell lines were used for the analysis. RNA-sequencing data of the Cancer Genome Atlas (TCGA) for skin cutaneous melanoma (SKCM) were obtained from Firehose using the Bioconductor package RTCGAToolbox (version 2.8.0). A provisional dataset of 469 samples were used for the analysis. Spearman rank correlation was used to assess correlation between gene expression levels.

Figure legends

Figure S1. *In silico* analysis of *IL13RA2* expression. Cancer Cell Line Encyclopedia (CCLE) was used to study the frequency of *IL13RA2* expression in various types of carcinoma cell lines.

Figure S2. Immunohistochemical analysis for IL13Rα2 expression in A375, A375-IL13RA2 KO, or A2058 xenograft and normal human tissues. (A) A375 xenograft (B) A375-IL13RA2 KO xenograft (C) A2058 xenograft, (D) testis, (E) stomach, (F) pancreas, (G) kidney, (H) lung. Scale bars: 50 µm.

Figure S3. Effect of IL-13 on the *in vitro* proliferation of various melanoma cells. In all, (A) 8 x 10^4 of SK-MEL-28, (B) 8 x 10^4 of SK-IL13Rα2 and (C) 1.8 x 10^4 of A375 cells, were seeded into 6-well plates and were grown in the presence or absence of IL-13. The cells were harvested after (A) 2, (B) 2 or (C) 3 days, respectively, and were counted. The number of cells at the indicated time period are expressed as mean ± SD. *$p < 0.05$; Student's t-test.

Figure S4. Effect of IL13Rα2 expression on tumour angiogenesis. Sections of tumours derived from the SK-MEL-28 (n = 6) and SK-IL13Rα2 (n = 5) cells were subjected to immunofluorescence staining with the anti-PECAM-1 antibody. Scale bar: 100 µm.

Figure S5. Correlation between *IL13RA2* and *AREG* mRNA expression levels in melanoma tissues. A scatter plot revealing the correlation between the *IL13RA2* mRNA and *AREG* mRNA expressions.

Figure S6. Effect of IL13Rα2 on VEGF expression in various melanoma cells. (A) The SK-MEL-28 melanoma cells were transfected with the IL13Rα2 expression vector, followed by qRT-PCR analysis to determine the expression of IL13Rα2 (top) and VEGF (bottom). (B) *IL13RA2* was knocked out in the A375 melanoma cells, followed by qRT-PCR analysis for the expression of IL13Rα2 (top) and VEGF (bottom). All values are mean ± SD. $*p < 0.05$; Student's t-test.

Figure S7. Full-length blots of cropped images shown in Figure 5A.

Supplementary Table 1. Summary of tissue microarray

Slide name	Position	Ratio of IL13RA2+ cells (%)	Organ	Pathological diagnosis
ME1004e	A10	10	Skin	Malignant melanoma of right sole
ME1004e	B2	15	Skin	Malignant melanoma of sole
ME1004e	C7	2	Skin	Malignant melanoma of heel
ME1004e	D8	90	Skin	Malignant melanoma of left thigh
ME1004e	D9	40	Vulva	Malignant melanoma of cunnus
ME1004e	H1	10	Lymph node	Metastatic malignant melanoma from armpit
ME1004e	E1	3	Skin	Malignant melanoma
ME1004e	E5	15	Skin	Malignant melanoma
ME1004e	F8	5	Skin	Malignant melanoma
ME1004e	G3	3	Skin	Malignant melanoma
ME1004e	A7	1	Skin	Malignant melanoma
ME208	D2	1	Skin	Malignant melanoma of left chest wall
ME208	D6	2	Skin	Malignant melanoma
ME208	H4	1	Lymph node	Metastatic malignant melanoma from armpit

Supplementary Table 2. Primers for quantitative RT-PCR

Transcript	Primer	Sequence (5’ to 3’)
GAPDH (GAPDH)	5’	GAAGGTGAAGGTCGGAGTC
	3’	GAAGATGGTGATGGGATTTC
ACTB (β-actin)	5’	TCACCCACACTGTGCCCATCTACGA
	3’	CAGCGGAACCGCTCATTGCCAATGG
IL13RA2 (IL13Rα2)	5’	TAAACCTTTGCCGCCAGTCT
	3’	AGGTCCCAAAGGTATGCTCC
AREG (amphiregulin)	5’	GTGTCCCAGAGACCGAGTTG
	3’	ATGGTTCACGCTTCCCAGAG
VEGFA (VEGF-A)	5’	AGGCCAGCACATAGGAGAGA
	3’	TTTCTTGCGCTTTCGTTTTT

Fig. S1

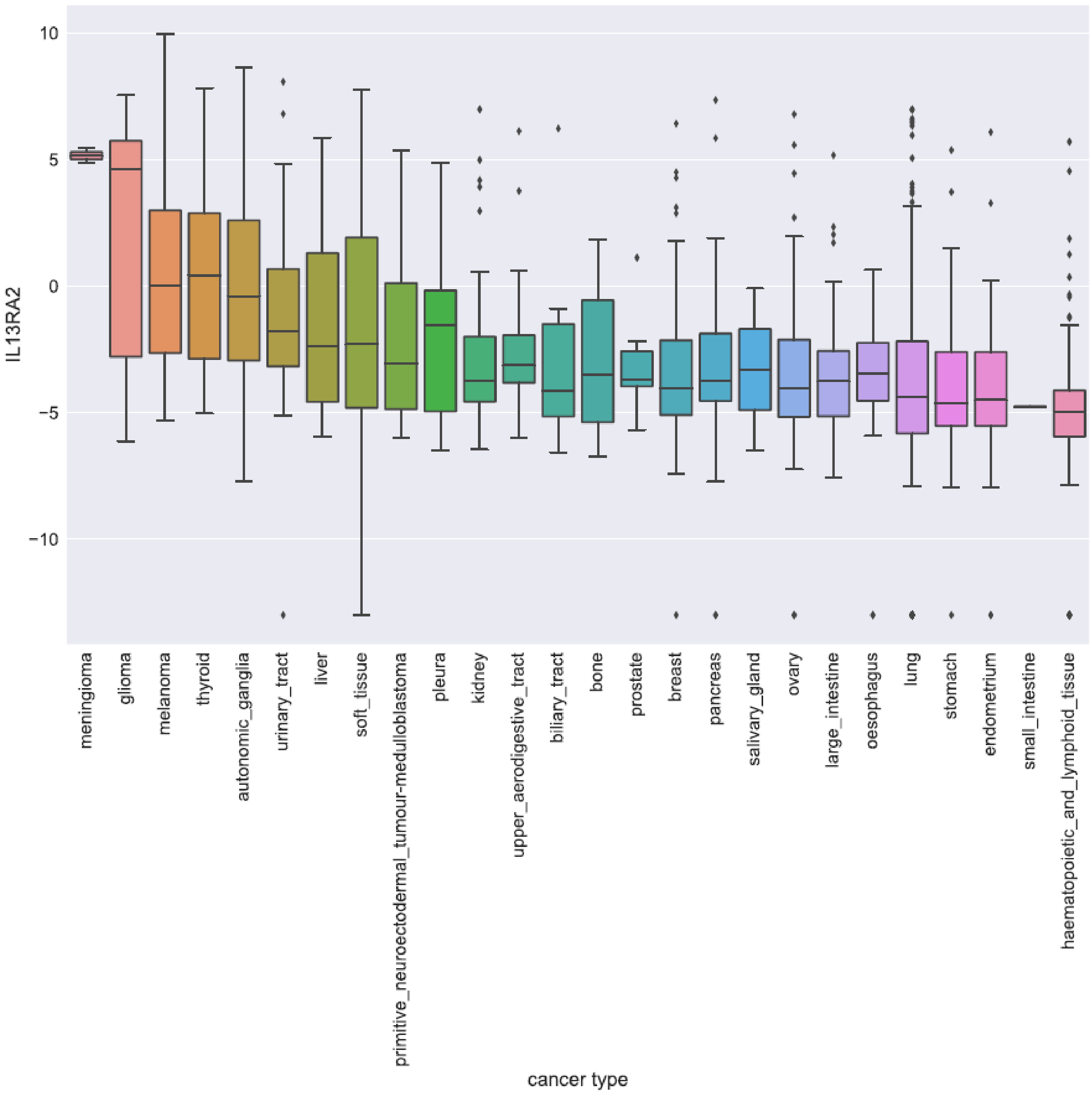
10
5
0
-5
−10
IL13RA2
meningioma
glioma
melanoma
thyroid
autonomic_ganglia
urinary_tract
liver
soft_tissue
primitive_neuroectodermal_tumour-medulloblastoma
pleura
kidney
upper_aerodigestive_tract
biliary_tract
bone
prostate
breast
pancreas
salivary_gland
ovary
large_intestine
oesophagus
lung
stomach
endometrium
small_intestine
haematopoietic_and_lymphoid_tissue
cancer type

Fig. S2

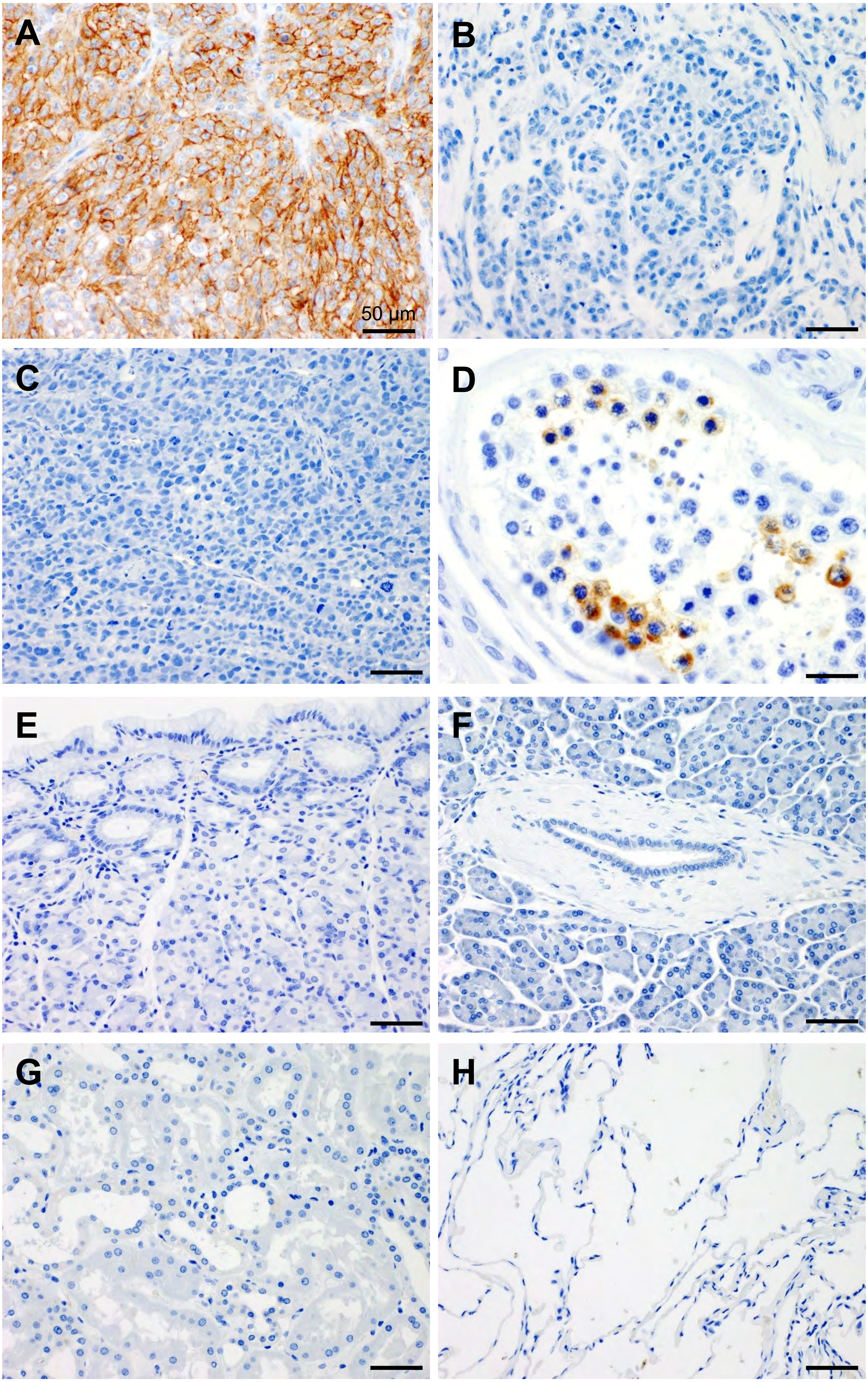

Fig. S3

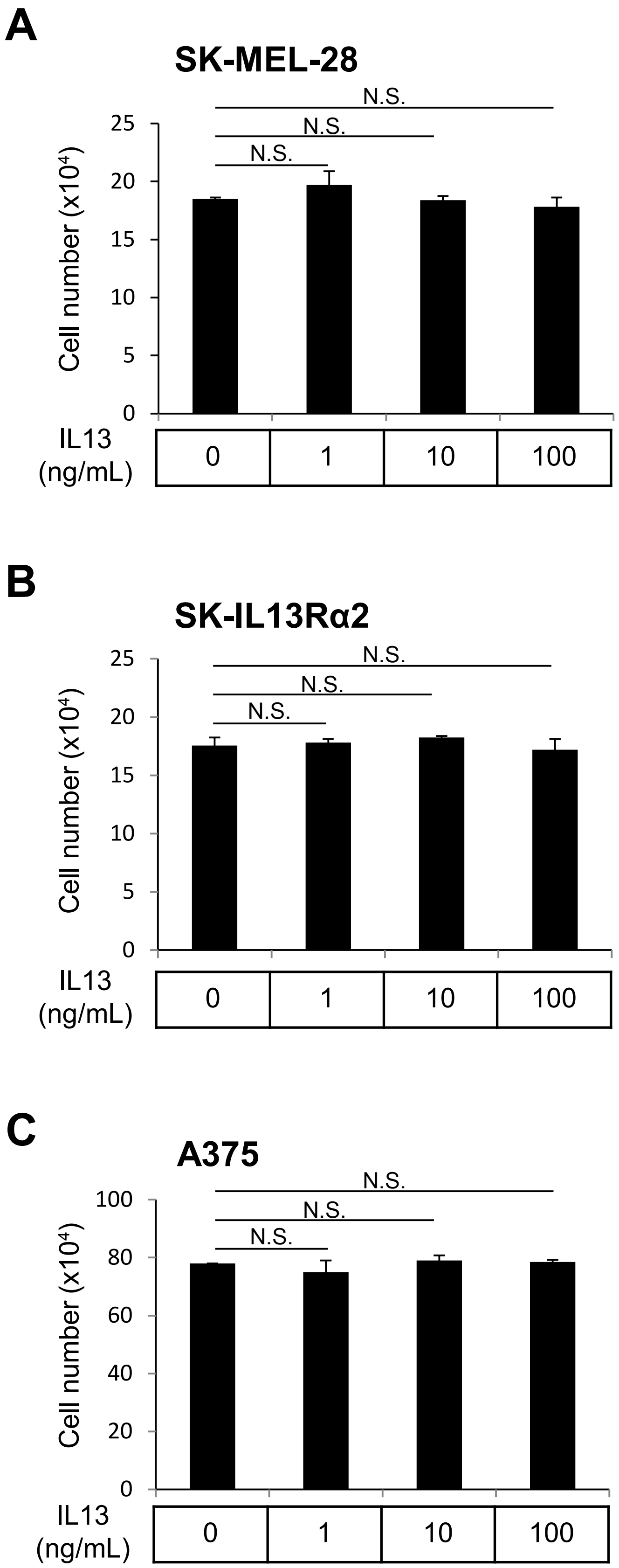

A
SK-MEL-28
N.S.
N.S.
N.S.
Cell number (x10⁴)
25
20
15
10
5
0
IL13
(ng/mL)
0
1
10
100
B
SK-IL13Rα2
N.S.
N.S.
N.S.
Cell number (x10⁴)
25
20
15
10
5
0
IL13
(ng/mL)
0
1
10
100
C
A375
N.S.
N.S.
N.S.
Cell number (x10⁴)
100
80
60
40
20
0
IL13
(ng/mL)
0
1
10
100

Fig. S4

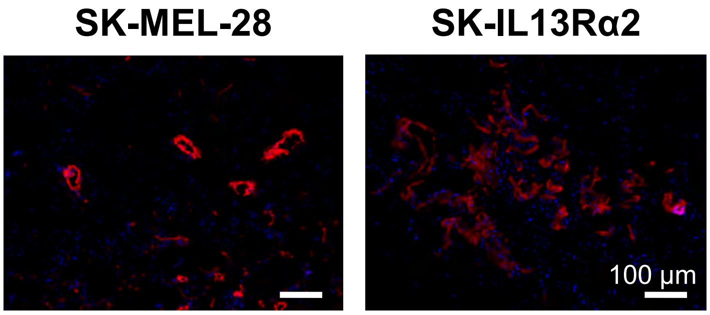

Fig. S5

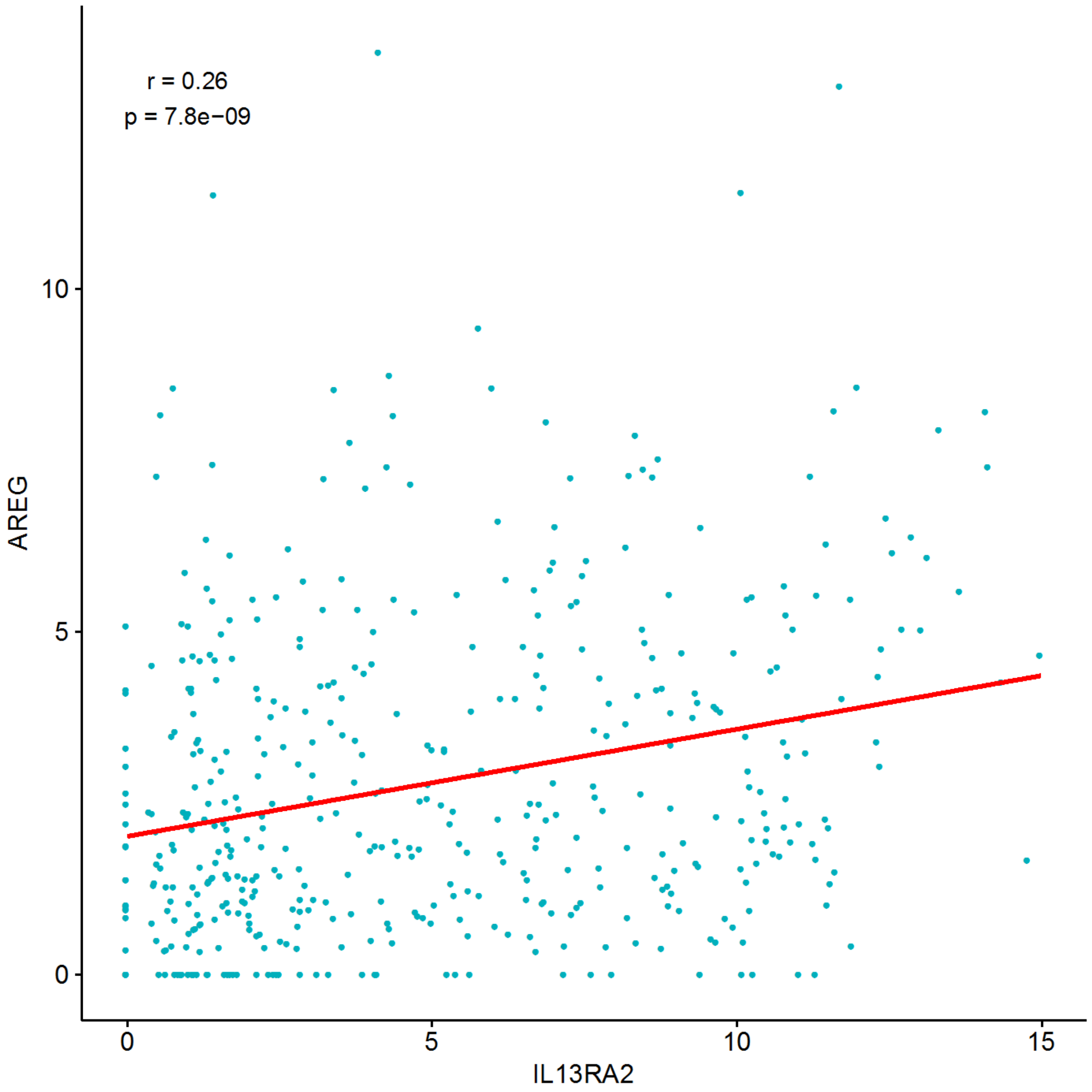

Fig. S6

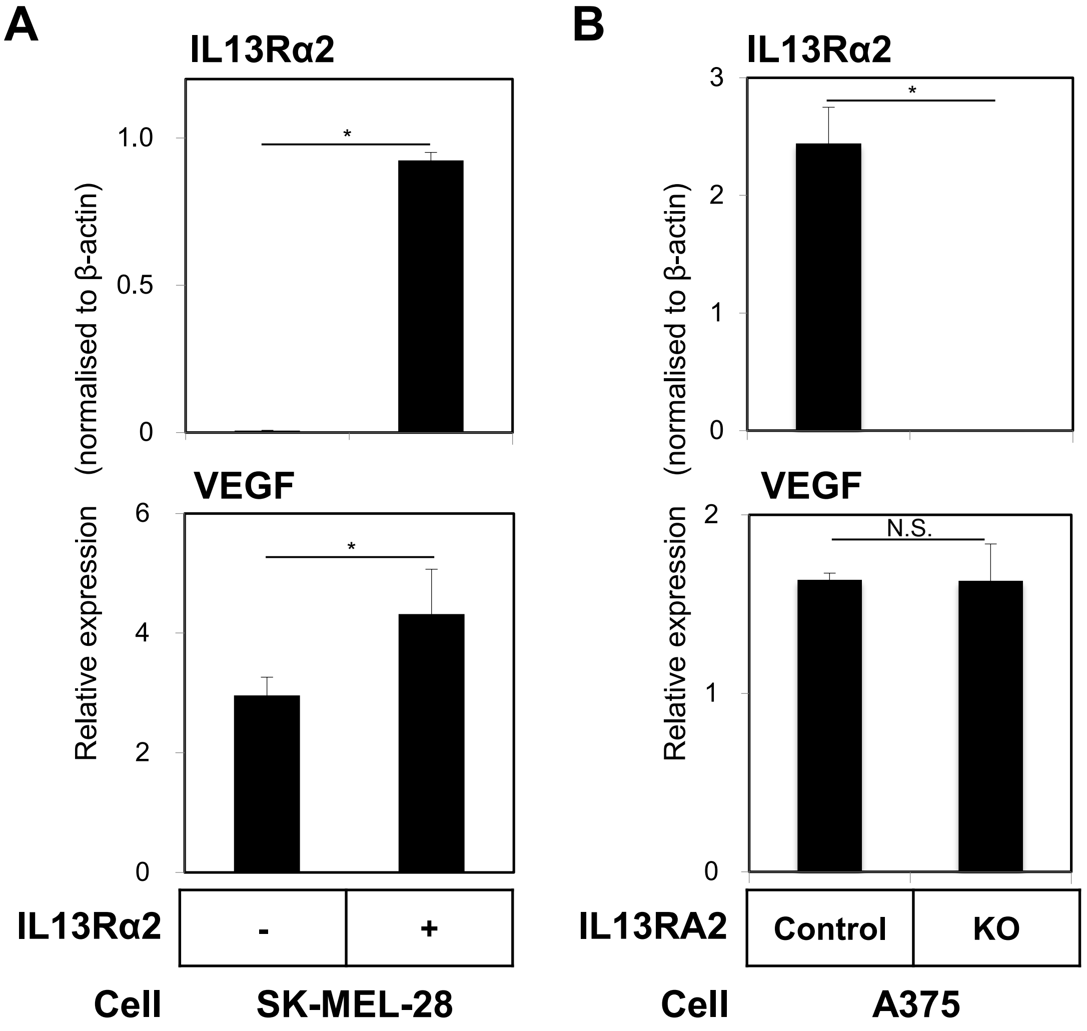

A
IL13Rα2
1.0
0.5
0
*
VEGF
6
4
2
0
*
Relative expression
(normalised to β-actin)
IL13Rα2
-
+
Cell
SK-MEL-28
B
IL13Rα2
3
2
1
0
*
VEGF
2
1
0
N.S.
Relative expression
(normalised to β-actin)
IL13RA2
Control
KO
Cell
A375

Fig. S7

IL13Rα2

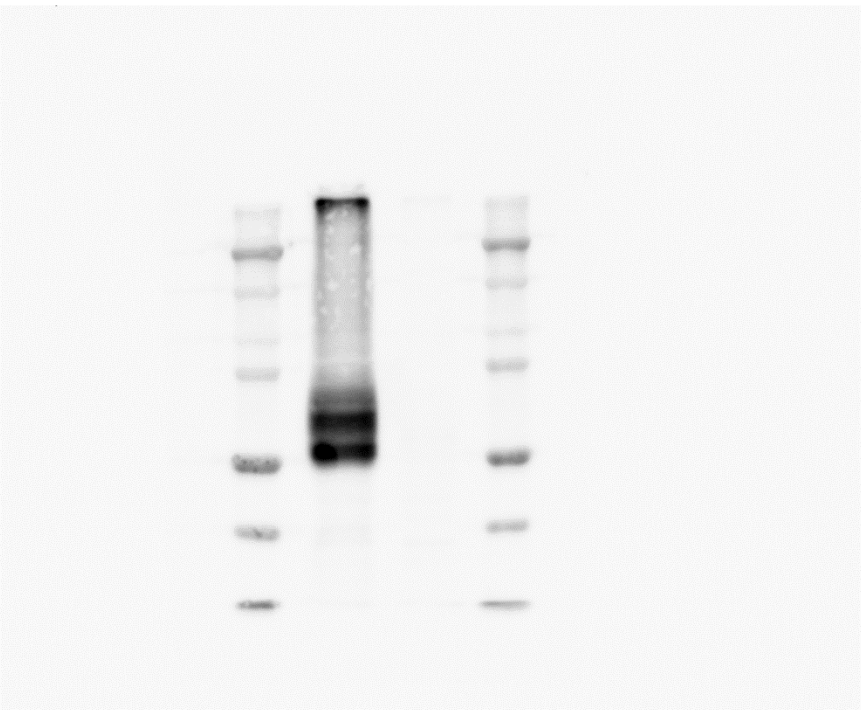

β-actin

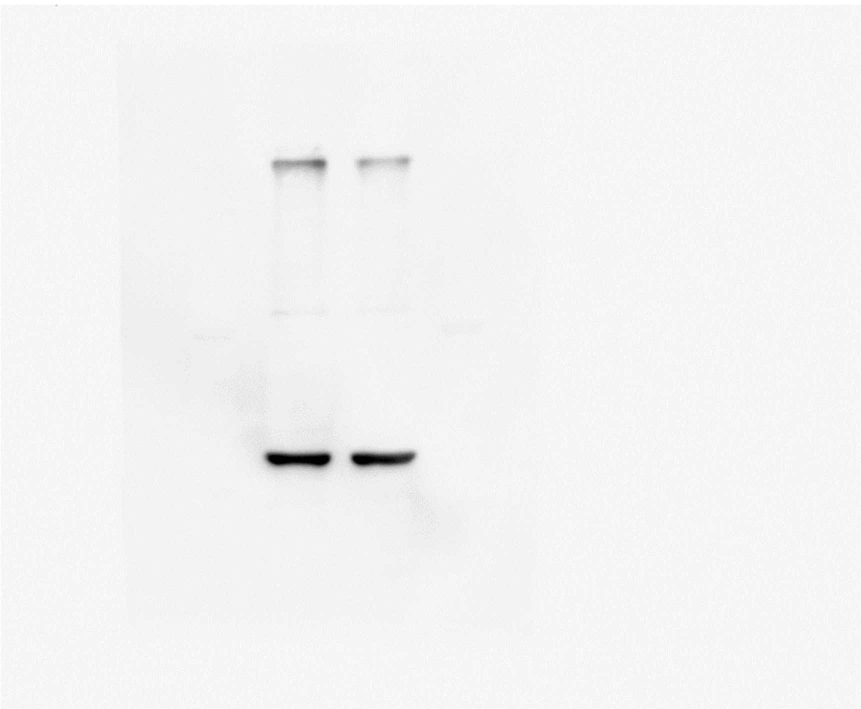

Supplement: Supplementary file 1 — Supplementary Information [file 41598_2019_39018_MOESM1_ESM.pdf]
